# Retraction: Circular RNA hsa_circ_0000467 modulates SGK1 to facilitate cell migration, metastasis, and EMT while repressing apoptosis in colorectal cancer by sponging miR-383-5p

**DOI:** 10.1039/d1ra90010b

**Published:** 2021-01-20

**Authors:** Laura Fisher

**Affiliations:** Royal Society of Chemistry, Thomas Graham House Science Park, Milton Road Cambridge CB4 0WF UK advances-rsc@rsc.org

## Abstract

Retraction of ‘Circular RNA hsa_circ_0000467 modulates SGK1 to facilitate cell migration, metastasis, and EMT while repressing apoptosis in colorectal cancer by sponging miR-383-5p’ by Chong Liu *et al.*, *RSC Adv.*, 2019, **9**, 39294–39303, DOI: 10.1039/C9RA07900A.

The Royal Society of Chemistry hereby wholly retracts this *RSC Advances* article due to concerns with the reliability of the data. The images in the article were screened by an image integrity expert who found that all the western blot bands have a very uniform shape that are unlikely to be genuine.

In addition, the paper was analysed by experts who fact-checked the identities of the described nucleotide sequence reagents,^1^ and found errors with the following nucleotide sequence reagents reported in the article: hsa_circ_0000467 forward and reverse primers, miR-383-5p forward and reverse primers and GAPDH forward and reverse primers. Therefore, the results shown in Fig 1, 2, 3, 4 and 7 are unreliable.

The authors were asked to provide the raw data for this article, but did not respond. Given the significance of the concerns about the validity of the data, and the lack of raw data, the findings presented in this paper are not reliable.

The authors have been informed but have not responded to any correspondence regarding the retraction.

Signed: Laura Fisher, Executive Editor, *RSC Advances*

Date: 7^th^ January 2021

## Supplementary Material
